# 

**Published:** 1831-09

**Authors:** 


### MONTHLY LIST OF MEDICAL BOOKS.

[*Medical Works cannot be entered on this List unless a copy be sent for the purpose, the titles of Books having frequently been sent to us as published which have not been published for weeks, or even months, after.*]

CHOLERA MORBUS. A short and faithful Account of the History, Progress, Causes, Symptoms, and Treatment of the Indian and Russian Cholera, taken from various authentic Sources; with Cases, as related by Practitioners in India; and also the interesting Case lately given to the Public by Dr. H. ROE, of the Westminster Hospital. By JOHN AUSTIN, Surgeon.—8vo. pp. 44. Hughes, London.

A Popular Treatise on the Teeth and Gums, and Diseases attendant on them; designed for the Use of Families. By JOHN WINCKWORTH.—4to. pp. 52; plates. Renshaw and Rush, London.

Elements of Practical Midwifery; or, Companion to the Lying-in Room. By CHARLES WALLER, Consulting Accoucheur to the London and Southwark Midwifery Institution; Lecturer on Midwifery and Diseases of Women and Children. Second Edition, with Additions.—18mo. pp. 200; plates. Highley, London.

Practical Observations on Prolapsus of the Rectum. By FREDERICK SALMON, F.R.C.S., author of a Practical Essay upon Contraction of the Rectum, Piles, &c.—8vo. pp. 105; plates. Whittaker, London.

Advice to the Public for the Prevention and Cure of the Asiatic Cholera. Translated from the German of Dr. J. R. LICHTENSTADT, Professor of Medicine, &c.—Souter, London.

Separation without Dissention. Observations addressed to General Practitioners, on the best Means of maintaining their Privileges and Respectability. By WM. COOKE, Surgeon.—Longman, London.

Medico-Chirurgical Notes and Illustrations. Part I. On some dangerous Affections of the Throat, which induce Sudden Death by Suffocation; on Strictures of the Œsophagus, and the Dangers of the Bougie; on the Cure of the Falling-down of the Bowel in Grown Persons; Anomalies in Rupture Operations, &c. By R. FLETCHER, Esq. Surgeon to the General Infirmary, Gloucester, &c.—4to. pp. 146; plates. Longman, London.

A Systematic Arrangement of British Plants; by W. WITHERING, M.D. Corrected and condensed; preceded by an Introduction to the Study of Botany, accompanied with Figures. By W. MACGILLIVRAY, A.M., Member of the Wernerian Natural History Society, &c.—8vo. pp. 390. Dove, London.

A Treatise on Auscultation, illustrated by Cases and Dissections. By ROBERT SPITTALL, lately Physician's Assistant, and now House Surgeon, Royal Infirmary of Edinburgh, &c.—8vo. pp. 280; plates. Grant, Edinburgh; and Hurst, London.

The Glasgow Medical Examiner. Nos. III. IV. and V.—Glasgow.

A Manual of Medical Jurisprudence, compiled from the best Medical and Legal Works: comprising an Account of, 1. The Ethics of the Medical Profession; 2. The Charters and Statutes relating to the Faculty; and 3. All Medico-legal Questions, with the latest Decisions: being an Analysis of a Course of Lectures on Forensic Medicine, annually delivered in London, and intended as a Compendium for the use of Barristers, Solicitors, Magistrates, Coroners, and Medical Practitioners. By MICHAEL RYAN, M.D. &c.—8vo. pp. 309. Renshaw and Rush, London.

### NOTICE.

*We shall be happy to insert the Critical Analysis of the Doctrines of Cullen, Clutterbuck, and Broussais, promised us by* "A Correspondent." *We deem it advisable not to publish the brief Remarks on Fever with which he has favored us: his peculiar opinions require a more detailed statement.*

ERRATUM. In the motto of the titlepage to the last volume, for "saluti" read "salutis."

